# The development of a comprehensive multidisciplinary care pathway for patients with a hip fracture: design and results of a clinical trial

**DOI:** 10.1186/1471-2474-15-188

**Published:** 2014-05-30

**Authors:** Elvira R Flikweert, Gerbrand J Izaks, Bas AS Knobben, Martin Stevens, Klaus Wendt

**Affiliations:** 1 University of Groningen, Department of Surgery-Traumatology, University Medical Center Groningen, PO Box 300001, Groningen 9700 RB, the Netherlands; 2 University of Groningen, University Center for Geriatric Medicine, Groningen, the Netherlands; 3 University of Groningen, Department of Orthopedics, University Medical Center, Groningen, the Netherlands; 4 Department of Orthopedics, Martini Hospital Groningen, Groningen, the Netherlands

**Keywords:** Care pathway, Elderly, Hip fracture, Fasting time, Length of stay

## Abstract

**Background:**

Hip fractures frequently occur in older persons and severely decrease life expectancy and independence. Several care pathways have been developed to lower the risk of negative outcomes but most pathways are limited to only one aspect of care. The aim of this study was therefore to develop a comprehensive care pathway for older persons with a hip fracture and to conduct a preliminary analysis of its effect.

**Methods:**

A comprehensive multidisciplinary care pathway for patients aged 60 years or older with a hip fracture was developed by a multidisciplinary team. The new care pathway was evaluated in a clinical trial with historical controls. The data of the intervention group were collected prospectively. The intervention group included all patients with a hip fracture who were admitted to University Medical Center Groningen between 1 July 2009 and 1 July 2011. The data of the control group were collected retrospectively. The control group comprised all patients with a hip fracture who were admitted between 1 January 2006 and 1 January 2008. The groups were compared with the independent sample t-test, the Mann–Whitney U-test or the Chi-squared test (Phi test). The effect of the intervention on fasting time and length of stay was adjusted by linear regression analysis for differences between the intervention and control group.

**Results:**

The intervention group included 256 persons (women, 68%; mean age (SD), 78 (9) years) and the control group 145 persons (women, 72%; mean age (SD), 80 (10) years). Median preoperative fasting time and median length of hospital stay were significantly lower in the intervention group: 9 vs. 17 hours (*p* < 0.001), and 7 vs. 11 days (p < 0.001), respectively. A similar result was found after adjustment for age, gender, living condition and American Society of Anesthesiologists (ASA) classification. In-hospital mortality was also lower in the intervention group: 2% vs. 6% (*p* < 0.05). There were no statistically significant differences in other outcome measures.

**Conclusions:**

The new comprehensive care pathway was associated with a significant decrease in preoperative fasting time and length of hospital stay.

## Background

A hip fracture has a strong negative effect on activities of daily living and consequently on quality of life. One year after the fracture, about 50% of patients are still more disabled than before the fracture, and during the first months after the operation the all-cause mortality rate rises up to eight-fold [1,2]. Although recent studies among Western populations report an age-adjusted decrease in incidence rates [3], the absolute number of hip fractures is expected to continue increasing in the coming decades because of the worldwide rise in life expectancy. In 1990 an estimated 1.7 million hips were fractured worldwide [4]. The estimated worldwide incidence of hip fractures for the year 2050 is 6.3 million [4].

In the USA expenditures for hip fractures are about one third of the total costs for all fractures and are rising faster than the general rate of inflation [4]. Besides the costs, hip fractures constitute a high burden on the medical and social system. The care for elderly patients with a femoral neck or pertrochanteric femoral fracture (further called hip fracture) is complex and challenging. Optimizing medical care is important, as treatment for a hip fracture is associated with significant mortality and morbidity. The increase in mortality rate persists beyond 10 years after the fracture, and only 25% of patients regain their prefracture ability to perform instrumental activities of daily living [5].

It is generally assumed that the high burden on the medical and social system can be lowered by developing multidisciplinary care pathways for older patients with a hip fracture. However, there is still no evidence that these pathways lead to better and more efficient care [6,7]. Most studies on care pathways for hip fractures investigate one specific component of care, such as care on the surgical ward or standard geriatric consultation [5,8-13]. For this reason we developed a comprehensive care pathway comprising the total care process, from arrival at the emergency room up to the end of the rehabilitation program.

The aim of this study was to investigate the first results of this new comprehensive care pathway for hip fractures, two years after implementation. Our main focus was on process outcome measures reflecting the effectiveness of the care pathway. We also examined patient related outcomes. Data of patients who were treated in the comprehensive care pathway were compared with data of a historical control group.

## Methods

### Design

A clinical trial in which the data of the intervention group were collected prospectively and compared with a historical control group. This study was conducted at the departments of Traumatology and Orthopedic Surgery of University Medical Center Groningen (UMCG). Informed consent was obtained of all patients in the intervention group. The study was approved by the Medical Ethical Committee of UMCG, which also approved the retrospectively access of the control group medical records.

### Subjects

#### Intervention group

The intervention group comprised all consecutive hip fracture patients who were admitted to UMCG between 1 July 2009 and 1 July 2011. Inclusion criteria were age 60 or older and hip fracture defined as a femoral neck fracture (dislocated or not dislocated) and pertrochanteric fractures (Arbeitsgemeinschaft für Osteosynthesefragen (AO) Comprehensive Classification 31.A.1; 31.A.2; 31.A.3). Exclusion criteria was severe multi-trauma (thoracic or abdominal). All patients were treated according to the protocols of the comprehensive care pathway. The data of the intervention group were collected prospectively.

#### Control group

The control group comprised all patients who were aged 60 or older and were treated at UMCG for a hip fracture between 1 January 2006 and 1 January 2008. They were treated following the standard care at that time; there was no multidisciplinary protocol involved. As a result, there was not a structured cooperation between the hospital and nursing homes and no strict discharge protocol. These data were retrospectively collected by case review and use of the electronic hospital registration system. No information was available about the stay in a nursing home.

### Intervention: comprehensive care pathway

**Figure 1:**
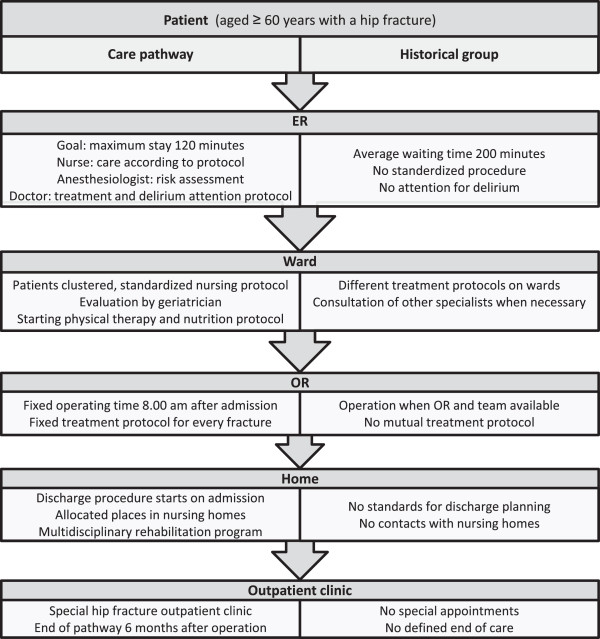
**Flow chart of the treatment in both groups.** Schematic representation of the differences between the treatment in the intervention group (comprehensive care pathway) and the historical control group (standard care). For details, see text. ER: emergency room, OR: operating room.

The comprehensive multidisciplinary care pathway was developed to include all elements of care in the trajectory, from arrival in the emergency room to the moment of discharge from the rehabilitation unit of the nursing home (Figure 1). Representatives of the departments of traumatology, orthopedics, geriatrics and anesthesiology developed the care pathway in close cooperation with representatives of the emergency department, the department of physical therapy and two nursing homes in the Groningen area. The core elements were:

#### Emergency room

**Figure 2:**
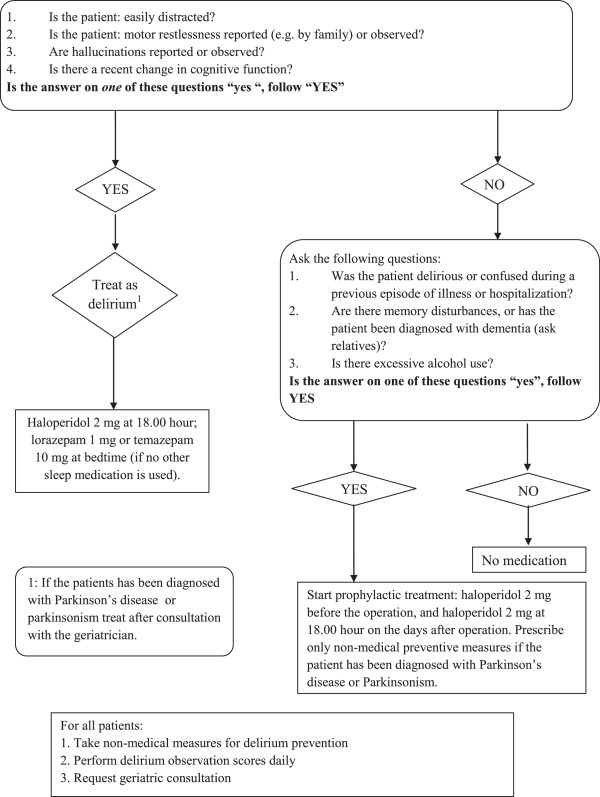
**Delirium protocol.** The delirium protocol that was part of the comprehensive care pathway and was applied upon arrival at the emergency room.

At the emergency room, when the diagnosis was clear, the care pathway started with an extensive nursing protocol. The most important part of this protocol was to transfer the patient to a bed with a pressure-relieving mattress as soon as possible. Other elements were to take blood samples and insert a short-term indwelling urinary catheter. The risk of postoperative delirium was estimated, using a standardized delirium protocol. Patients at high risk were prescribed pharmacological prophylaxis as well as non-pharmacological measures (Figure 2). Also, a perioperative anesthesiological risk assessment was made in the emergency room. The decision whether other specialists had to be consulted was coordinated by the anesthesiologist in order to limit the number of doctors per patient.

#### Planning of surgery

Patients with a hip fracture were enrolled for surgery at a fixed time in the emergency operating rooms (OR). Each morning at 8:00 a.m. an emergency OR was available for a patient with a hip fracture. An experienced surgeon and OR team were also available. There was a uniform treatment protocol for both the surgery teams of the department of orthopedics and the department of traumatology. Because of the fixed time of surgery, 8:00 a.m. on the day after admission, patients were allowed to eat until midnight – eight hours before surgery – causing minimal discomfort.

#### Clustering of patients

All hip fracture patients were clustered on one nursing ward in order to increase the knowledge of the nursing staff and, thus improve the quality of care for the patient, with extra attention for care for elderly patients (early start of rehabilitation, adequate diet, pressure ulcer prevention). Additional attention was paid to delirium and its prevention, by applying the Delirium Observation Screening scale (DOS) by nurses on each shift [14,15].

#### Geriatric consultation service

During the stay at the nursing ward the patients were routinely visited by the geriatrician on a daily basis.

#### Discharge procedures

A strong focus was set on discharge procedures. Upon arrival at the hospital the patient was registered for one of the two participating nursing homes. For purposes of efficient patient transfer, at the nursing homes beds were reserved for hip fracture patients. In addition, the doctor at the nursing home was able to view the data of patients with a hip fracture in the data management systems of UMCG. Since this doctor handled the admissions, he could follow the progress of the patients and anticipate at their arrival.

#### Outpatient clinic

Patients were seen at the outpatient clinic at six weeks and three and six months after surgery. A standardized work-up at the fracture and osteoporosis outpatient clinic took place within six weeks after surgery. If necessary, the patient was examined at the fall prevention outpatient clinic.

### Outcome measurements

Demographic characteristics, medical history, fracture characteristics and trauma mechanism were registered at the emergency room. The American Society of Anesthesiologists (ASA) classification [16] and the type of implant were recorded at the operation theatre.

To get an impression of the effectiveness of the care pathway, process outcome measures were determined both in the intervention and control group: duration of the emergency room (ER) stay, preoperative fasting time, waiting time for operation, use of general analgesia, treatment with prosthesis, consultation of other medical specialist, length of hospital stay, and destination upon discharge were taken from the electronic hospital registration system after discharge. In addition patient related outcome measures were gathered in both groups after discharge to evaluate the effectiveness of the care pathway on other relevant outcomes: occurrence of complications, occurrence of delirium, in-hospital mortality, 30-day postoperative mortality and need for reoperation within one year.

Patients were asked to visit the outpatient clinic at six weeks, three months and six months postoperatively. If applicable, admission time and discharge location for the nursing home in the intervention group were provided as well as date and cause of death.

### Statistical analyses

Statistical analysis was performed with IBM SPSS Statistics 19.0 (IBM, Amonk, NY). Descriptive statistics were used to describe the main characteristics of the population. For continuous variables, the intervention and control groups were compared with the independent sample t-test or, if appropriate, the Mann–Whitney U-test. For categorical data, the Chi-squared test (Phi test) was used. The effect of the intervention on fasting time and length of stay was adjusted by linear regression analysis for differences between the intervention and control group at baseline. The dependent variable in the regression model was the natural logarithm (log_e_) of fasting time (hours), or the natural logarithm of admission time (days). The log-transformation was done because the distribution of both variables was skewed. The effect of the intervention on 30-day mortality was adjusted by logistic regression analysis. The dependent variable in this analysis was death within 30 days of operation (yes/no). In all regression models, the independent variables were intervention group (yes/no), age (years), female (yes/no), living in a nursing home (yes/no) and ASA ≥3 (yes/no). The level of statistical significance was set at 0.05.

## Results

### Baseline characteristics

The intervention group included 256 patients (32% men and 68% women) with a mean age (SD) of 78 (9) years. At the time of the fracture, 27 patients (11%) were living in a nursing home, 180 (70%) were living independently (alone or with others), and 49 (19%) in a home for the aged. A total of 133 patients (52%) were able to walk without aids, 72 others (20%) were used to walking aids outside as well as inside their homes, and the remaining patients (28%) used walking aids only for longer distances . The control group included 145 patients (28% men and 72% women). They were slightly older than the intervention group (mean (SD) 80 (10) years, *p* = 0.22), but there were no statistically significant differences in other demographic characteristics between the two groups (Table 1).

**Table 1:** Baseline characteristics of the intervention and control group

	**Intervention group**	**Control group**	**p**
Number	256	145	n.a.
Women (%)	174 (68)	104 (72)	0.84^b^
Mean age, years (SD)	78 (9)	80 (10)	0.22^a^
Living in nursing home (%)	27 (11)	15 (10)	0.87^b^
Median ASA classification (IQR)	3 (2–3)	3 (2–3)	0.52^b^
Fracture type (%)			
Femoral neck	142 (55)	83 (57)	0.57^b^
Trochanteric	114 (45)	62 (43)	

*Abbreviations:* n.a: not applicable; SD: standard deviatiation; IQR: interquartile range.

^a^independent sample t-test; ^b^Chi-squared test (Phi test).

Median ASA classification (interquartile range, IQR) of the included patients in both groups was 3 (2–3). In the intervention group 32 patients (13%) had no relevant medical history; 16% of the patients did not take medication. In the control group these numbers were 12% and 9% respectively, with no statistically significant difference. In 55% of the cases in the intervention group there was a femoral neck fracture, in the remaining cases there was a trochanteric fracture. There was no statistically significant difference in fracture type between the two groups (Table 1).

### Outcome measures

#### Process outcome measures

Preoperative fasting time and length of hospital stay were significantly lower in the intervention group (Table 2). After adjustment for differences in potentially confounding factors at baseline (age, gender, ASA classification and living situation), fasting time in the intervention group was 34% shorter than in the control group (Table 3). The number of patients who had to wait more than one day for the operation was also lower in the intervention group than in the control group: 8% vs. 14% (*p* < 0.05). Length of stay was 21% shorter in the intervention group (Table 3).

**Table 2:** Outcome measures in the intervention and control group

	**Intervention group**	**Control group**	**p**
Number	256	145	
**Process outcome measures**			
Mean time on ER (SD), minutes	196 (75)	208 (87)	0.59^a^
Median fasting time (IQR), hours	9 (8–12)	17 (8–24)	<0.001^a^
Waiting > 1 day for operation (%)	20 (8)	20 (14)	0.042^b^
General analgesia (%)	166 (65)	73 (50)	0.006^b^
Treated with a prosthesis (%)	92 (36)	46 (32)	0.44^b^
Consult of other specialists (%)	43 (17)	42 (29)	0.05^b^
Median length of stay (IQR), days	7 (6–10)	11 (7–16)	<0.001^a^
Discharge to nursing home (%)	195 (76)	100 (69)	0.88^b^
**Patient outcome measures**			
Patients with complications (%)	130 (51)	71 (49)	0.76^b^
Patients with delirium (%)	42 (16)	20 (14)	0.48^b^
In-hospital mortality	5 (2)	8 (6)	0.03^b^
Mortality in 30 days (%)	13 (5)	13 (9)	0.78^b^
Reoperation within 1 year (%)	31 (12)	23 (16)	0.29^b^

*Abbreviations:* SD: standard deviation; IQR, interquartile range.

^a^independent sample t-test; ^b^Chi-squared test (Phi test).

**Table 3:** The effect of the comprehensive care pathway on fasting time, admission time and 30-day mortality

	**Intervention group**	**Control group**	**Crude ratio**		**Adjusted ratio**^**a**^	
				**Ratio**	**95% CI**	**P**
Median fasting time, hours	9	17	0.53	0.66	0.60-0.73	<0.001^b^
Median admission time, days	7	11	0.64	0.79	0.70-0.88	<0.001^b^
30-day mortality,%	5	9	0.56	0.56	0.25-1.30	0.18^c^

^a^Adjustment was made for age, gender, living condition and ASA classification.

^b^Regression analysis (ANOVA), ^c^logistic regression analysis.

The only significant difference in the operative procedure was the more frequent use of general instead of spinal anesthesia in the care pathway group (Table 2). Overall, 138 (34%) of the patients were treated with a prosthesis and 263 (66%) with a osteosynthesis (in 125 (31%) cases a dynamic hip screw was used, in 104 (26%) cases an intramedullary nail and in 34 (8%) cases cannulated screws). There was no significant difference in the percentage of patients treated with a prosthesis between the groups.

Apart from the standard liaison geriatrician, other medical specialists (mostly cardiologists) were consulted for 17% of the patients in the intervention group and 29% of the patients in the control group (*p* = 0.05).

Most patients in both groups were transferred to a nursing home, and only 24% in the intervention group and 31% in the control group were directly discharged to their own home. In the intervention group the median length of stay at the nursing home was 7 weeks, whereupon 63% of the patients were discharged to their own homes. The mortality during the stay in the nursing home was 8%.

#### Patient outcome measures

The incidence rate of complications did not differ between the intervention and control groups (Table 2). The most frequent complication was delirium, which was diagnosed in 16% of the intervention group and 14% of the control group (*p* = 0.48). Other frequently encountered complications were infections and cardiac problems.

The in-hospital mortality rate was significantly lower in the intervention group than in the control group: 2% vs. 6% (*p* = 0.03). However, there was no statistically significant difference in the mortality rate after 30 days: 5% vs. 9% (*p* = 0.78). When adjustment was made for potential confounders, the 30-day mortality in the intervention group was 44% lower, but this difference was not statistically significant (Table 3).

## Discussion

In this study we found that it is possible to introduce a comprehensive care pathway for patients with hip fracture and improve the outcomes for this category of vulnerable patients in a short period of time. The most important result of the comprehensive care pathway was a significant reduction in preoperative fasting time and in length of hospital stay.

Fasting time is not often reported in the literature as an independent variable. However, it was recently found that a fasting time of more than 12 hours in a patient with a hip fracture is an independent risk factor for in-hospital complications and mortality [17]. Fasting time is an important outcome measure and it is likely that a reduction in fasting time improves postoperative recovery. A reduction in length of hospital stay after introduction of a care pathway for hip fracture was found in several other studies [7,18-21]. Although there was a considerable heterogeneity in length of stay, almost all studies report a hospitalization twice as long than reported in our study [19-22]. Probably this was caused by the fact that the care pathway in this study was comprehensive and involved a close relationship with the nursing home.

No change was found in the time spent in the emergency room. In our opinion, an ER stay of more than three hours can be considered as too long. The lack of improvement on this point might be explained by many different interests at the emergency room. Compared to other necessary changes, reduction in length of stay for a specific group had a low priority. At the same time, however, the care for patients with hip fracture in the emergency room had improved. Upon arrival at the emergency room, all patients were transferred to a hospital bed with a pressure-relieving mattress instead of lying on a stretcher all the time, which was common practice in the control group. Furthermore, the anesthesiologist performed the preoperative work-up in the emergency room and coordinated the consultations with other specialists. This last measure and the fact that a geriatrician visited every patient on the ward probably led to a reduction in the number of doctors per patient.

Patients in the intervention group had general anesthesia more often. Although previous studies report a better outcome for patients who are operated under spinal analgesia [23,24], a more recent study did not show a difference in delirium risk for patients who were operated under regional or general anesthesia [25]. In this study there was no association between type of anesthesia and complications or mortality either.

The complication rate was similar in both groups. However, for the control group these data were collected retrospectively from the patient files whereas for the intervention group they were registered prospectively in an electronic database. It is therefore likely that the complication rate was underestimated in the control group. Although we think that a complication rate of 50% is still very high, several other (prospective) studies report similar complication rates [21,26,27]. Comparable to these other studies, we found that delirium, infections and cardiac problems were the most frequent complications in older patients with hip fracture.

Several studies report a significant decrease in the risk of delirium after introduction of geriatric consultation and one study demonstrated a reduction in severity and duration of the delirium after starting haloperidol prophylaxis [7,9,13,28]. For this reason, both interventions were included in the comprehensive care pathway that we developed. Remarkably, the comprehensive care pathway was not associated with a lower risk of delirium. Here too, retrospective collection of the data could have caused an underestimation of delirium risk in the control group. However, it is also possible that improvements in usual care due to the growing general awareness of the risk of delirium in recent years led to a relative low overall incidence of delirium in our study.

The in-hospital mortality rate decreased significantly, to 2%. Most studies report a higher in hospital mortality in this population [18,26], but Vidan [21] reports a rate as low as 0.6%. Still, because of the short hospitalization it will be more reliable to use the 30-day mortality. There was a decrease in 30-day mortality, yet this was not significant in regression analysis.

A major limitation of the present study is its design as a clinical trial with historical controls. Because of this design, the data collection in the two groups is not uniform, which can influence the outcome measures. As discussed above, this probably caused underestimation of some important outcome measures such as incidence of postoperative complications and delirium. This does not weaken our findings though. On the contrary, it strengthens them, as the comprehensive care pathway is probably more effective than observed in this study. Furthermore, it is unlikely that the design negatively influenced the measurement of more robust outcomes such as preoperative fasting time and length of hospital stay.

Our study also has some strengths. All the professionals who were involved in the development of the comprehensive care pathway highly valued its multidisciplinary nature. At regular intervals, the implementation of the care pathway was evaluated with representatives of all medical and paramedical specialties that were part of the pathway. This resulted in a smooth implementation of the comprehensive care pathway and a high adherence to its protocols.

## Conclusions

This new comprehensive care pathway for patients with hip fracture significantly reduced preoperative fasting time and length of hospital stay. These results need to be confirmed in a randomized controlled trial.

## Competing interests

The study has had a grant, as part of a greater research project, from Biomet® and Trauma Center Northern Netherlands, a special department of the University Medical Center. These organizations however, do not have any influence on the design nor publications of the study.

The grant is used to pay part of the personnel costs.

## Authors’ contributions

EF designed the study, collected the data, analyzed the data, interpreted the results, and wrote the manuscript. GI designed the study, interpreted the results and was co-writer of the manuscript. BK designed the study and reviewed the manuscript. MS designed the study, interpreted the results and was co-writer of the manuscript. KW designed the study, reviewed the manuscript and arranged the finances. All authors read and approved the final manuscript.

## Pre-publication history

The pre-publication history for this paper can be accessed here:

http://www.biomedcentral.com/1471-2474/15/188/prepub
